# An assessment of option B implementation for the prevention of mother to child transmission in Dschang, Cameroon: results from the DREAM (Drug Resource Enhancement against AIDS and Malnutrition) cohort

**DOI:** 10.11604/pamj.2016.23.72.7958

**Published:** 2016-03-10

**Authors:** Anna Maria Doro Altan, Francis Taafo, François Fopa, Ersilia Buonomo, Maria Cristina Marazzi, Karin Nielsen-Saines, Stefano Orlando, Paola Scarcella, Fausto Ciccacci, Sandro Mancinelli, Massimo Magnano San Lio, Leonardo Palombi

**Affiliations:** 1 DREAM programme, Community of Sant'Egidio, Rome, Italy; 2 DREAM Center, Dschang, Cameroon; 3 Department of Biomedicine and Prevention, University of Rome Tor Vergata, Italy; 4 University LUMSA, Rome, Italy; 5 Department of Pediatrics, David Geffen UCLA School of Medicine, Los Angeles, California

**Keywords:** HIV, mother-to-child transmission (MTCT), breastfeeding, Cameroon, Option B

## Abstract

**Introduction:**

Scaling up of antiretroviral therapy (ART) to HIV+ pregnant women is crucial for the elimination of HIV infection in children. The aim of this study was to evaluate the feasibility and effectiveness of triple ART for Prevention of Mother-to Child Transmission (PMTCT) in Cameroon.

**Methods:**

HIV-positive pregnant women attending the DREAM Centre of Dschang, Cameroon for prenatal care were enrolled in a prospective cohort study, and received ART until the end of breastfeeding or indefinitely if their CD4 count was <350mm^3^. Infants were evaluated for HIV infection at 1, 6 and 12 months of age.

**Results:**

A total of 298 women were enrolled. Among them, 152 were already on established ART. Women were followed until 6 months after delivery with a retention rate of 92.6%. Eight women died. Those with a CD4 count <350 cells/mm^3^ during pregnancy had the highest mortality risk (RR 2.53; 95% CL= 1.86-3.44). The HIV transmission rate was 1.2% at 12 months with an HIV free survival of 91%. In the proportional Cox regression analysis, the following factors were positively associated with infant mortality: maternal CD4< 350 cells/mm^3^, no breastfeeding in the first 6 months of life, weight-for-age z score<-2.

**Conclusion:**

Results confirm the feasibility and effectiveness of the implementation of Option B, with very low rates of HIV MTC transmission, and potential benefits to the health of mothers and infants with earlier initiation of ART. Breastfeeding again demonstrates to be highly beneficial for the growth and survival of HIV exposed children.

## Introduction

It is well known that administration of triple antiretroviral therapy (ART) to HIV infected women during pregnancy and breastfeeding can reduce HIV mother to child (MTC) transmission to less than 5% [1–4]. In Sub-Saharan Africa, the use of ART for prevention of MTC transmission (PMTCT) has been limited until recent years with various single drug regimens and short course dual antiretroviral regimens applied. In 2013, the WHO endorsed the use of lifelong ART (Option B+) or ART until at least the end of breastfeeding (Option B) for HIV PMTCT [5]. Many challenges remain in sub Saharan African countries in order to achieve the goal of elimination of MTC transmission: insufficient access of pregnant women to HIV testing and treatment, poor implementation of PMTCT programs, and insufficient levels of retention in care [6–8]. Cameroon is among the 22 priority countries for the elimination of HIV mother-to child transmission. HIV+ rates among pregnant women have been estimated at 7.8% at a national level [9]. Cameroon is implementing since 2012 a gradual shift to Option B+ strategy for prevention of MTC transmission. There are few studies in Cameroon describing the use of triple ART for PMTCT [10, 11]. Nutritional strategies are also very important in order to guarantee a better chance of survival to HIV exposed children. Some studies show that children born to HIV infected mothers often have a higher mortality than children born to uninfected mothers, and worse nutritional status [12–14]. In HIV exposed uninfected children, this is partially due to suboptimal feeding strategies. The effectiveness of ART in reducing postnatal transmission through breastfeeding has to a large extent resolved the dilemma of how HIV-infected mothers should feed their infants. Some studies report reductions in mortality in association with breastfeeding in HIV exposed uninfected infants [15]. DREAM is a program of care for people with HIV designed and managed by the Community of Sant'Egidio, which is now working in ten African countries including Cameroon. The DREAM program offers free of charge state-of-the-art treatment, diagnostic facilities and nutritional supplementation to patients. The DREAM programme has been implementing PMTCT with triple antiretroviral therapy since 2004 in Mozambique and Malawi [16, 17]. In 2008 we proposed to the Ministry of Health of Cameroon the implementation of a study to evaluate the feasibility and outcomes of Option B implementation in the district of Dschang, a mixed urban/rural area in the western region of the country. Results are helpful in the planning and implementation of further scaling up. The proposal was accepted in 2009 under the framework of an operational research project approved by the Ministry of Health. The aim of the present study was to evaluate HIV transmission rates, as well as infant and maternal mortality, retention in care, and HIV-free infant survival. Infant growth in the first year of life was also evaluated.

## Methods

**Study design:** prospective cohort study.

**Study setting:** the study was conducted at the DREAM Centre, Hospital St Vincent de Paul, Dschang, West Region, Cameroon.

**Population:** the study population included all HIV-positive pregnant women attending the DREAM Centre in Dschang from September 2009 to September 2011. Women were referred from the surrounding antenatal care (ANC) centres, or self-referred as seeking care for HIV.

**Procedures and measurements:** pregnant women already on established ART at presentation continued on treatment. Women not on ART initiated lifelong ART as soon as the 14^th^ week of gestational age was reached if they had a CD4 count of less than 350 cells/mm^3^. Women presenting with a CD4 count greater than 350 cells/mm^3^initiated ART at 25 weeks of gestational age, and discontinued ART after 6 months postpartum. Women were counselled to exclusively breastfeed, to introduce complementary food when the child reached 5 months of age, and to wean the child at the age of 6 months. Women, who did not want/could not breastfeed, were counselled to give exclusive formula milk until 6 months of age. ART regimens consisted primarily of: Zidovudine+Lamivudine+Nevirapine (AZT+3TC+NVP) or Stavudine+Lamivudine+Nevirapine(D4T+3TC+NVP), the main first line regimens used at that time in the country. Women who experienced NVP-related reactions were shifted to a regimen containing Lopinavir/Ritonavir. Women were offered: monthly health checks, weight and height measurements, adherence counselling, medical visits, CD4 counts every 6 months, baseline and yearly viral load (VL) monitoring, pre-ART and routine whole blood count and biochemistry, nutritional counselling and supplementation depending on nutritional status. Infants were offered: single dose Nevirapine at birth, monthly checks, weight, length/height and arm circumference measurements, medical visits, and were evaluated for HIV infection by branched DNA assay at 1 and 6 months of age. At 12 months a rapid test was performed, and if positive a virological test was performed. A rapid HIV test was performed also at 18 months of age. Height and weight were measured according to standard anthropometric methods and z scores were calculated with the software “ANTHRO” having as reference the WHO Child Growth Standards 2006 [18]. Underweight was defined as having a weight for age z score (WAZ)<-2 and stunting as having a length-for-age z score (LAZ)<-2. Women or children not presenting to the scheduled appointments were called by the staff. Women and children not returning after 3 months were considered lost to follow up. Women asking a note of transfer for another facility were registered as transferred.

**Statistical analysis:** descriptive data are presented as means with standard deviations (SD), or median and IQ ranges. Differences between means were tested with the Student t test. Categorical data were compared using the chi-square test or the Fisher test as appropriate. The Kaplan-Meier method was used to estimate the rates of child survival (including HIV-free survival) and Cox proportional hazards model was used to identify factors associated with mortality in children. For all statistical tests, two sided P values of less than 0.05 were considered to be statistically significant. For women, data were censored at 6 months after delivery and for children at 12 months of age. SPSS v. Win 18.0 was used for data analysis.

**Ethics statement:** the study was approved by the National Ethic Committee (133/CNE/SE/09) and authorized by the Ministry of Health (Aut 631, 2009). An informed consent was signed by all participants.

## Results

**Figure 1:**
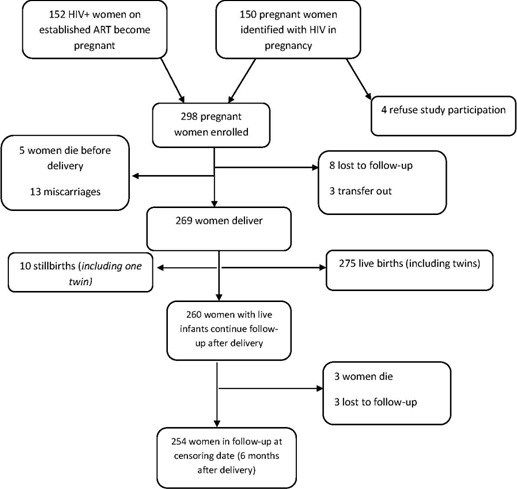
Women cohort profile

**Study participant characteristics:** a total of 298 women were enrolled in the study over the two year period. Among them, 152 were already on established ART. Of 146 pregnant women who initiated ART during pregnancy, 81 (55.5%) had a CD4 count>350 cells/mm3. The mean age was 30.24±5.4 years. The main clinical data and the cohort profile are summarized in Table 1 and Figure 1. At the time of counselling, 70% of women reported knowledge of the serological status of their partner.

**Table 1:** Characteristics of women in the study

		All	A. Initiated ART in pregnancy, CD4>350/mm^3^	B. Initiated ART in pregnancy, CD4<350/ mm^3^	C. Established ART before pregnancy	T test (p-value)
**Number**		298	81	65	152	
**Baseline data**	Age (years), mean±SD	30.2±5.4	29.4±6.3	30.0±5.3	30.8±5	A vs Bp=0.5 A vs C p=0.1 B vs C p=0.3
	CD4 (cells/mm3), mean±SD	443±298	578±230	236±87	460±165	A vs B p<0.001 A vs C p<0.001 B vs C p<0.001
	Haemoglobin (g/dl), mean±SD	11.4±1.3	11.2±1.1	10.6±1.2	11.9±1.2	A vs B p=0.03 A vs C p<0.001 B vs C p<0.001
	CV log	2.46±1.8	3.53±1	4.08±0.95	1.23±1.46	A vs B p=0.002 A vs C p<0,001 B vs C p<0,001
	Arm Circumf. (cm), mean±SD	28 ±3.3	28.1±3.2	27±2.9	28.4±3.4	A vs B p=0.03 A vs C p=0.4 B vs C p=0.004
	BMI, median (IQR)	26.45 (24.17-29.3)	26.7 (24.8-29.3)	25.5 (23.3-28.4)	26.7 (24.2-29.5)	
**Pregnancy outcomes**	No. of Abortions	13	1	1	11	
	No. of Stillbirths	10	3	3	4	
	No. live births	260	71	57	132	
	Duration ARV before delivery (days), median (IQR)	292 (94-802)	93 (69-104)	105 (64-154)	810 (567-1110)	
	Delivered at hospital	190	55	37	98	
	Delivered at home	10	3	2	5	
	Delivered at health centre	60	14	18	28	

**Maternal outcomes:** at the censoring time point (6 months after delivery), 8 women (2.6%) had died, 11 (3.7%) had abandoned the programme, 3 had been transferred to other facilities due to proximity reasons, and 276 (92.6%) had completed the protocol. LTFU rates were 5.5% for women presenting to care during pregnancy and 2% for women on established ART prior to pregnancy. Seven of the 8 maternal death occurred in women with a CD4 count of less than 350 cells/mm^3^ (RR 2.53; 95% CL= 1.86-3.44). Two hundred seventy five living infants were born (including twins). Only 3.6% of women delivered at home, 73.1% delivered at the hospital and 22.9% at a health centre facility. The median duration of ART before delivery was 94 days (IQR 65.5-110) for women initiating ART in pregnancy, and 801 days (IQR 567-1110) for women already on established ART. We observed 13 abortions and 10 stillbirths (Table 1).

**Table 2:** Hazard risk for mortality in children (cox proportional hazards model, stepwise)

	Hazard Risk	CL 95%	P value
**Maternal Cd4 <350/mm** ^**3**^	3.8	1.20-8.48	0.021
**Underweight**	4.36	1.68-11.28	0.002
**Breastfeeding**	0.039	0.13-0.95	0.039

**Figure 2:**
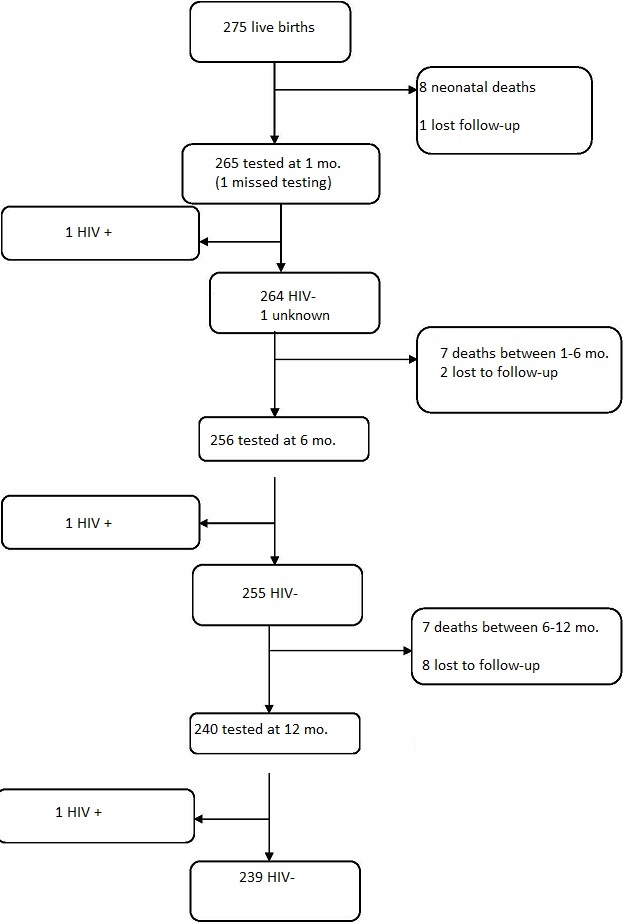
Children cohort profile

**Mother-to-child HIV transmission:** HIV status was available for 265 of 275 infants (96.3%) at one month of age, and for 242 infants at 12 months of age (88% of the original cohort). At 12 months, 3 infants had acquired HIV, corresponding to a transmission rate of 1.2%. The HIV transmission rate was 1.5% among children born to women who initiated ART in pregnancy, and 0.8% among women on established ART (difference not statistically significant, p=0.53). One infant was found to be infected at one month, one at 6 months and one at 12 months of age. The three children at the censoring date were receiving ART and in good health. The infant cohort profile is shown in Figure 2. LTFU rates among children were 5 per 100 person years. Eight of 11 LTFU happened after six months of age.

**Figure 3:**
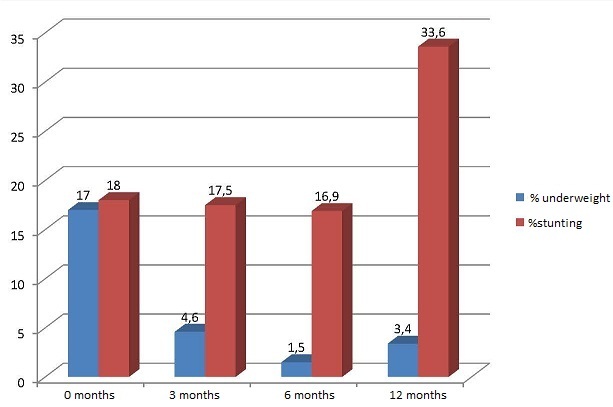
Percentage of underweight and stunted children in the first year of age

**Figure 4:**
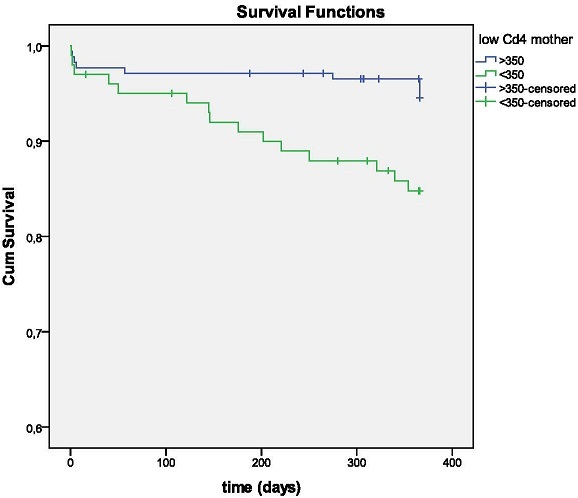
Infant survival according to maternal CD4 count

**Child growth and survival:** forty-five infants(17%)out of 275 were born with a birth weight of less than 2.5 kg. A positive correlation was observed between birth weight and length of ART in mothers before delivery: women in the lowest quartile of duration of ART gave birth to children with a birthweight slightly lower than women in the highest quartile of ART duration (2.8±0.56 kg against 3.0±0.55 kg; p=0.023). Eighty-nine percent of infants (243 out of 272 for whom the information was available) were exclusively breastfed until 5 months of life. Mean WAZ and LAZ are summarized in Figure 3. Twenty-two infants died before one year of age, corresponding to an infant mortality of 80 per 1000 (neonatal mortality of 29 per 1000, post neonatal of 51 per 1000) (Figure 4). HIV-free survival was 91% at 12 months. In the proportional Cox regression analysis, the following factors were positively associated with infant mortality (Table 2): maternal CD4<350 cells/mm^3^, no breastfeeding in the first 6 months of life, WAZ<-2 at any time from birth to 12 months.

## Discussion

The present study reports our results regarding the feasibility and the effectiveness of triple ART for HIV PMTCT in pregnant women followed in DREAM Centre in Cameroon. We included both women starting ART during pregnancy and women already on established ART who became pregnant. As countries are expanding access to ART and moving to Option B+, there will be an increasing number of women in this last situation. In our cohort, women on established therapy had better clinical and laboratory values (in terms of haemoglobin levels and nutritional conditions) as compared to women starting ART during pregnancy, regardless of CD4 cell count, and relatively high CD4 values overall (mean 460±165). HIV transmission rates were extremely low in the established therapy group (0.8% at 12 months of age) even if no statistical difference emerged when comparing both groups of mothers. The significantly and substantially higher level of mortality observed in women with a CD4 cell count of less than 350 cells/mm^3^ during pregnancy (RR 2.53; 95% CL= 1.86-3.44) is an important confirmation that implementation of the new criteria of WHO Consolidated Guidelines [5] (initiation of treatment in pregnancy irrespective of CD4 cell count or initiation of treatment at a CD4 cell count<500) is a key strategy not only for prevention of HIV in infants, but also as a strategy for optimization of maternal survival. The maternal retention rate was 92.6% six months after delivery, including deaths, LTFU and transfers. Other studies carried out in sub Saharan Africa report disengagement from care varying from 30% to 50% [19, 20]. Data from the implementation of Option B+ in Malawi showed 77% retention after 12 months at a national level [21]. Other studies have shown that the highest attrition occurs in the first months between testing and linkage to care [22]. The service delivery model of the DREAM Centre, with the PMTCT program embedded into the ART program, likely has a beneficial effect on retention, as shown in some studies [23], although we have no information on women lost between testing and linkage to care. Other reasons for the high rates of retention observed are timely initiation of ART and the active monitoring and adherence reinforcement strategy approach of the DREAM Programme, previously described [24].

The proportion of children born with a low birthweight was 17% in our study, which is higher than data for the general population in Cameroon [25] but comparable to data from other studies of infants born to HIV+ mothers in the country [11, 26]. Some past studies have expressed concern regarding the potential impact of ART treatment on low birth weight [27], especially if PI containing regimens are used [28]. Many other authors did not observe such correlation [29, 30]. On the other hand, the adverse effects of HIV infection of itself on nutritional condition are well known. Although we did not perform a comparative analysis between women using/not using ART, in our cohort we did not observe an association between longer duration of ART and low birth weight. On the contrary, women with the longest exposure to ART before delivery gave birth to newborns with slightly higher birth weights (2.8 Kg vs. 3Kg respectively; p=0.023). The HIV transmission risk in our cohort was very low, and HIV free survival was comparable to data obtained in other studies on the effectiveness of maternal triple ART in other Sub Saharan countries [31]. A retrospective study carried out in Yaoundé on 418 HIV+ pregnant women [11] reported HIV transmission rates of 6.6% at 6 weeks. In this study women were using different drug regimens, with only 17.5% using a triple drug regimen. Another study carried out in Yaoundé with 587 HIV+ pregnant women [10], found transmission rates of 1.7% among women on triple antiretroviral therapy, comparable to those obtained in the present study. Eighty-nine percent of the women chose exclusive breastfeeding, this rate is higher if compared to data from Yaoundé and Eastern Cameroon [8, 32] and can be related both to the rural residency of the population and to the effectiveness of counselling, given the updated knowledge of health staff on risks and advantages of breastfeeding within the context of HIV infection. The nutritional status of children was good, with 4.6% infants being underweight at 3 months and 1.5% at 6 months, although a decline in mean WAZ and HAZ was observed from 6 to 12 months, the period in which infants were weaned. A similar decline has been observed in other studies, particularly when infants stop being breastfed [33]. In particular, at 12 months 33.6% of children were stunted, which is consistent with observations made in another study in the same area [34]. These rates are likely to improve with the prolongation of breastfeeding to 12 months, as recommended by the WHO [35]. The child mortality in our cohort was 80 per 1000. HIV transmission seemed to have a relatively low impact on HIV-free survival, as in our cohort all deaths occurred among HIV-exposed uninfected infants, although since HIV testing was not performed at birth, it is not possible to exclude an association between neonatal mortality and in utero HIV transmission. As in other studies [30], maternal baseline CD4 cell count of less than350/mm^3^ was a predictor of infant mortality. Other significant predictors in the multivariate analysis were a low weight for age score at any age, including birth, and not being breastfed. Breastfeeding was shown to be highly beneficial to infant growth and survival, as shown in previous studies [36]. Limitations to the study include the relatively small sample size of women and children, the lack of complete information on adherence, and the absence of early HIV testing at birth, which precluded us from determining HIV in utero transmission rates.

## Conclusion

The study confirmed the feasibility and substantial advantages in the implementation of triple antiretroviral therapy for HIV PMTCT in Cameroon, not only for effective prevention of HIV vertical transmission but also for reduction of maternal mortality. Our results indicate that full implementation of the new WHO consolidated guidelines for HIV PMTCT will have a very favourable outcome in this population. Extending breastfeeding while mothers receive ART until at least 12 months of life will likely positively affect the nutritional status and reduce mortality among HIV exposed infants.

### What is known about this topic

- Triple antiretroviral therapy to HIV infected women during pregnancy and breastfeeding (Option B, B+) can reduce HIV mother to child transmission to less than 5%.
- Insufficient levels of retention in care are among the obstacles for elimination of MTC transmission.
- HIV exposed infants have worse nutritional status than HIV unexposed infants.

### What this study adds

- Experience of option B in Cameroon.
- Data on clinical and biological parameters on women already on ART and having a pregnancy.
- In this cohort of HIV exposed infants, maternal CD4, absence of breastfeeding and low weight for age are independent predictors of infant mortality.
